# The Use of a Visual Scene Display for the Acquisition of Manding in Young Children with Complex Communication Needs

**DOI:** 10.3390/bs16081384

**Published:** 2026-08-12

**Authors:** Elizabeth R. Lorah, Sheida K. Raley, Madison Maddox, Whitney P. Moser, Christine Holyfield, Stephen MacNeil, Cynthia Zastudil

**Affiliations:** 1 Curriculum & Instruction, University of Arkansas, Fayetteville, AR 72701, USA; sheida@uark.edu (S.K.R.); mpmaddox@uark.edu (M.M.); wcprice@uark.edu (W.P.M.); 2 Communication Sciences, University of Arkansas, Fayetteville, AR 72701, USA; 3 Computer Science, Temple University, Philadelphia, PA 19122, USA; stephen.macneil@temple.edu (S.M.);

**Keywords:** augmentative and alternative communication, artificial intelligence, complex communication needs

## Abstract

The purpose of the study was to evaluate the use of an artificial intelligence (AI)-powered visual scene display (VSD) prototype, with a time delay, least-to-most prompting, and reinforcement in the acquisition of a mand repertoire for three preschool-aged children with complex communication needs (CCN), including those with suspected or confirmed autism diagnoses. A non-concurrent multiple baseline design across participants was used to evaluate the effects of the VSD on independent manding. All three participants’ results suggest increases in independent manding following the introduction of the intervention. Brianna met the mastery criterion within nine intervention sessions, while Nick and Vera met the mastery criterion within nine and thirteen intervention sessions, respectively. Maintenance data suggested continued independent manding for two of the three participants following intervention. Results are consistent with previous research supporting the use of VSDs with young children with CCN and extend this literature by providing preliminary evidence for the use of AI-powered VSDs to support mand acquisition. Limitations of the prototype technology, clinical implications, and future directions are discussed.

## 1. Introduction

For many autistic children, speech alone does not meet their daily communication needs. In fact, due to the lack of diverse communication supports across environments, by age nine, 48% of autistic children either have no or few spoken words, or do not speak in complete sentences (Chapin et al., 2022). For autistic children who also have complex communication needs (CCN; i.e., children with limited or no access to verbal speech and are unable to use speech to meet daily communication needs), augmentative and alternative communication (AAC) systems such as high-tech speech-generating devices (SGD) can be used to support their communication repertoires. With early and effective intervention and access to appropriate communication supports, autistic children with CCN can develop meaningful communication skills that promote social relationships, self-determination, employment opportunities, and active participation in their communities throughout adulthood (Holyfield et al., 2025; Light et al., 2025; McNaughton et al., 2025). To address the environmental barriers children with CCN experience, Skinner’s (1957) seminal text Verbal Behavior can serve as a foundational framework for designing communication supports for young autistic children with CCN (Tincani et al., 2020).

Skinner’s (1957) taxonomy of primary verbal operants is often applied to the body of research that exists for communication interventions using AAC as a primary support system for people with CCN (Lorah et al., 2022). Using Skinner’s elementary “verbal operants”, verbal behavior can be classified as a mand when behavior is under the control of a motivating operation such as hunger or pain, and in which the response specifies the reinforcer. For example, a child says “ball” and is given a ball. An echoic is verbal behavior in which the behavior has 1:1 correspondence with the antecedent stimuli that occasions the behavior, and the consequence is generalized conditioned reinforcement. For example, a parent says “ball”, the child says “ball”, and the parent responds “you’re right! It is a ball!” A tact is a verbal operant that is evoked by a non-verbal stimulus, and the consequence is generalized conditioned reinforcement. For example, a child sees a ball rolling down towards him, and he says “Ball!” and the parent responds “Yes, that is a ball!” Finally, an intraverbal is a verbal operant in which the response is evoked by prior verbal behavior, but there is no 1:1 correspondence with the prior verbal behavior; and the consequence is generalized conditioned reinforcement. For example, a parent says, “take me out to the…” and the child responds, “ball game…” (Lorah et al., 2022).

Within Skinner’s (1957) behavioral framework, the mand is the only verbal operant that directly benefits the speaker with access to tangible, desired stimuli, often unconditioned reinforcers (Sundberg & Michael, 2001). A mand is controlled by what an individual wants. Mands receive reinforcement specific to the particular mand. For example, if a child mands for a cookie, for the behavior to be classified as a mand (versus another operant), the listener must provide the speaker with a cookie (Sundberg & Michael, 2001). Given this unique quality of the mand, it is often the first operant targeted when AAC is introduced to young children with CCN, and as such, there is a robust literature base to support the use of AAC in mand acquisition (Lorah et al., 2022).

Furthermore, the importance of a mand repertoire can be directly linked to self-determination. Causal Agency Theory defines self-determination as a dispositional characteristic manifested as acting as the causal agent in one’s life, whereby people make things happen in their lives in ways that are aligned with their preferences, interests, values, and goals (Shogren & Raley, 2022; Shogren et al., 2015). Although self-determination is widely recognized as an important predictor of positive outcomes across the life course (e.g., Mazzotti et al., 2021), comparatively little research has examined how to intentionally build self-determination for young children with CCN (Didion et al., 2021). For young children with CCN, communication is one of the earliest and most meaningful mechanisms through which they can express preferences, make choices and decisions, request desired outcomes, and influence the actions of others in their circles of support. Consequently, when opportunities to develop and use a functional mand repertoire are limited, children may have fewer opportunities to communicate preferences and influence their environments, potentially thwarting opportunities to build self-determination and, in turn, contributing to reduced quality of life both in childhood and across the life course. For young children with CCN, developing a functional mand repertoire through AAC may represent one of the earliest opportunities to experience autonomy and agency. By requesting desired items, activities, assistance, or social interaction, children can influence outcomes that matter to them and participate more meaningfully in decisions affecting their lives. As such, mand instruction may create opportunities that are theoretically aligned with the development of self-determination.

To better support the communication of young autistic children who use AAC, visual scene displays (VSDs) may be used as an alternative to or to supplement traditional grid-based displays on the screen of an SGD (Lorah et al., 2026). VSDs are personalized, context-rich images that reflect activities, people, items, or events that are relevant to the speaker. When using an SGD, a VSD may be displayed on a tablet and include interactive “hotspots” that generate spoken words, sounds, or text when selected, allowing the user to communicate within a given context (Patenaude et al., 2025). For example, if a young child is playing at the park, there could be a picture of the playground structure on the tablet with interactive hotspots for the “slide”, “swing”, and “climber”. When the speaker selects the hotspot for “slide”, the listener can provide the associated reinforcement for the speaker’s behavior.

One benefit of the VSD is that it is highly personalized and contextually relevant to the user. Personalized, predictive, and responsive to environmental context are qualities of AAC that adult AAC users described as important for AAC systems to incorporate. Adult AAC users further described the need for AAC systems that adapted to them rather than those systems that required their behavior to adapt to it, the AAC system (Lorah et al., 2022). VSDs may offer a way to integrate these qualities into formative communicative experiences for young children with CCN. From a Causal Agency Theory perspective, self-determination builds through the interaction between a person’s capacities and the opportunities, supports, and contexts available to them (Shogren & Raley, 2022). Because VSDs are grounded in personally meaningful experiences and embedded within naturally occurring contexts, they may create opportunities for children to communicate about activities, people, and events that matter to them. In doing so, VSDs may support children in expressing preferences, directing interactions, and influencing outcomes within their environments, all of which are foundational aspects of acting as a causal agent in one’s life and being self-determined.

For example, if a young child with a CCN could take a picture of an item or activity they want to engage in or discuss, take the picture with their SGD, and then AI could label that item or activity for the user, using computer vision. Recently, the integration of AI into AAC systems is making those dreams of AAC users (Lorah et al., 2022) a reality. The use of computer vision and generative AI may offer young children with CCN the ability to create their own VSD, even if they are pre-literate. With computer vision, a picture of a playground structure with a hotspot drawn on the “slide” can use generative AI to produce the digitized output “slide”, similar to those VSD apps that do not use AI but rather require operational assistance by a literate adult or support person recording the output or speaking the word into the VSD for the user. In other words, the young child with a CCN could take a picture of an item or activity they want to engage in or discuss, take the picture with their SGD and then AI could label that item or activity for the user. AI-supported VSDs may eventually support more personalized communication opportunities. In particular, by empowering children to create communication supports that reflect their interests, activities, and experiences, AI-supported VSDs may provide opportunities for children to build agency and communicate about what matters most to them. In this way, AAC may be more responsive to the child’s goals and priorities rather than relying exclusively on communication opportunities identified by others (e.g., an adult, other support person). The AI-powered VSD prototype evaluated in this study explores this potential in that it used computer vision and generative AI to label the targeted item and produce the digitized output of the SGD.

While an AI-powered VSD would enhance the experiences of a young child with CCN in terms of their ability to create their own communication options, as a first step, a proof-of-concept study is necessary to address this research gap and ensure that such a prototype could be used for mand acquisition following the construction of the VSD. Therefore, this paper seeks to address the extent to which an AI-powered VSD prototype, implemented with time delay, least-to-most prompting, and reinforcement, facilitates the acquisition of manding in young children with CCN. Throughout this manuscript, the term “AI-powered VSD intervention” refers to the multicomponent instructional package evaluated in this study, consisting of the AI-powered VSD prototype implemented in conjunction with time delay, least-to-most prompting, reinforcement, and clinician mediation. Accordingly, the present study was not designed to isolate the effects of the AI component independent of the accompanying instructional procedures.

## 2. Method

### 2.1. Participants

As indicated in Table 1, participants included three preschool-aged children with CCN. Participants were recruited for the study based on the following criteria: (a) a diagnosis of autism spectrum disorder from an independent agency, or suspected autism and currently on the waitlist for a diagnosis; (b) no history of independent AAC use; (c) fewer than ten spoken words; (d) level one mand and echoic repertoires as measured by the Verbal Behavior Milestones Assessment and Placement Program; and (e) were between the ages of two and five-years old. Two females, Brianna and Vera, and one male, Nick, participated in the study. Informed consent was obtained from all three participants’ parents prior to the onset of the study. Brianna was four years old and was diagnosed with autism by an independent agency. She primarily used single-word vocalizations and gestures to communicate. She did not have a history of using an AAC prior to the onset of the study. Vera was two years old with suspected autism. At the time of the study, she was on a waitlist for a formal diagnosis. She primarily used one-to-two-word vocalizations and gestures to communicate and also had no history of using an AAC prior to the onset of the study. Nick was three years old with a diagnosis of autism from an independent agency. He primarily used single-word vocalizations and gestures to communicate; he had some history with using an AAC prior to the onset of the study, but never used it independently.

### 2.2. Setting and Materials

The setting for this study was a preschool classroom for young children with CCN, housed in a speech-language clinic at a major university in the Southeastern Conference. Sessions took place in a designated therapy room within the clinic, where the participants and the researcher were either seated side by side at a table or near each other on the carpet. The material included a prototype of an AI-powered VSD generator that was loaded on a 13-inch Apple iPad Pro^®^ (Apple Inc. 2025, Cupertino, CA, USA). The prototype was developed by the team for research rather than commercial purposes. The AI powered the SGD to produce individualized visual scene displays based on the participant-specific photographs and vocabulary targets. Once a photo was captured, the system used AI computer vision technology to generate vocabulary (i.e., nouns, verbs, etc.) based on stimuli in the photograph within two seconds after the photograph was captured. Once a vocabulary word was selected by the adult who mediated the identification of the vocabulary, a hot spot was drawn on the screen of the device with a swipe or touch; the vocabulary word would attach to the stimuli in the hotspot. Thus, the prototype evaluated in the present study was an adult-mediated AI-assisted VSD rather than a child-generated communication display. This allowed for personalized and immediately deployable communication displays for each participant occurring within three seconds of the capturing of the photograph. The computer vision AI model was 100% accurate in identifying the stimuli photographed. The synthetic voice output was also 100% accurate. Once the hotspot was activated with a touch, it produced a synthetic voice output that matched the photograph captured in the VSD. For example, if a picture of available art materials was taken, a hotspot was drawn around the crayons in the display, and that hotspot was then activated, a voice output of “crayons” would be generated for this initial proof-of-concept evaluation.

During mand training sessions, the iPad was positioned within three inches of the participant, consistent with the procedural protocol described in the general procedures section. Additional materials included preferred toys and activities identified for each participant through a free operant preference assessment (Cooper et al., 2020) conducted prior to the onset of the study. During the assessment, the participants were observed engaging with toys and activities within the preschool classroom. Data were collected on the duration and frequency of interactions with items. Those same items were presented to the participant at the start of each mand training session as an informal, in vivo preference assessment. Preferred items were used to contrive motivation during both baseline and intervention sessions and were delivered as reinforcement following correct responses. Paper data sheets were used to record participant responses (Independent or Prompted) across all trials during both baseline and intervention sessions.

### 2.3. Design

The design was a non-concurrent multiple baseline design across participants (Watson & Workman, 1981). A non-concurrent multiple baseline design was selected because it allows researchers to demonstrate a functional relation between the independent and dependent variables through the systematic, staggered introduction of intervention across participants while accommodating the practical constraints of applied clinical settings in which participants may not begin intervention simultaneously. This design was appropriate for the current study because participants entered the preschool clinic setting at different points in time, allowing intervention to be introduced without unnecessarily delaying instruction for participants who met predetermined decision-making criteria.

The design was implemented across three preschool-aged participants: Brianna, Vera, and Nick. Each participant initially completed five baseline sessions during which opportunities to mand for preferred items were contrived using the visual scene display (VSD); however, no prompting procedures were provided during baseline. Following baseline, Brianna was introduced to the intervention phase, during which model prompting procedures were implemented to support independent manding. Brianna was required to meet the predetermined mastery criterion of at least 80% independent responding across three consecutive sessions before the intervention was introduced to Vera. Once Vera met mastery criteria, the intervention was introduced to Nick. The staggered introduction of the intervention across participants allowed for the replication of treatment effects across three demonstrations, strengthening preliminary evidence for the increase in manding following the introduction of the AI- powered VSD intervention.

### 2.4. Researchers

Five interventionists were involved in the implementation of the study procedures. The primary investigator was a faculty member in Special Education at the university where the study took place and was also a Board-Certified Behavior Analyst-Doctoral™ (BCBA-D). The secondary investigator was a master’s-level student enrolled in an applied behavior analysis program at the university who was accruing supervised fieldwork hours toward eligibility for the Board-Certified Behavior Analyst (BCBA) examination. The remaining interventionists included two doctoral students in Curriculum and Instruction who were also Board-Certified Behavior Analysts and served as practicum supervisors within the university research clinic, as well as one master’s-level student enrolled in the practicum clinic and studying applied behavior analysis. The same interventionist worked with each participant throughout the study, with the exception of the primary investigator, who worked with each of the participants. In general, two interventionists were paired with a participant, with one conducting the training sessions and the second collecting data; however, during IOA sessions both interventionists collected data.

The primary investigator trained all secondary interventionists on the study procedures prior to the onset of the study. Training included instruction on baseline procedures, intervention procedures, prompting procedures, data collection methods, and reinforcement procedures. Secondary interventionists were required to demonstrate procedural implementation with 100% fidelity prior to independently conducting study sessions. Ongoing supervision was provided by the primary investigator throughout the duration of the study to ensure consistency in the implementation of procedures across interventionists.

### 2.5. General Procedures

The procedures of this study were approved by the university’s Institutional Review Board and included standard educational practices. All sessions were conducted within the speech-language clinic classroom by a member of the research team described above. Each participant received two sessions per therapy day, with a total of five trials conducted within each session, resulting in ten trials per therapy day. Two sessions per day were selected to maximize learning opportunities while remaining appropriate for the age and developmental levels of the participants.

Following the non-concurrent multiple baseline design, all participants initially completed five baseline sessions. During baseline, opportunities to mand for preferred items were contrived using individualized visual scene displays (VSDs); however, prompting procedures were not implemented. Following the completion of five baseline sessions, Brianna was introduced to the intervention phase while Vera and Nick remained in baseline. During intervention, model prompting procedures were implemented following opportunities for independent responding. Participants remained in intervention until they met the predetermined mastery criterion of at least 80% independent responding across three consecutive sessions. Once Brianna met mastery criteria, Vera was introduced to intervention procedures while Nick remained in baseline. Following Vera’s achievement of mastery criteria, Nick was introduced to intervention procedures. This process continued until all participants had been exposed to the intervention phase. Data were collected on each trial using a paper data sheet. Interventionists recorded the target item and whether the participant emitted an independent response or prompted/no responses.

Baseline. During baseline, the participant was observed interacting with preferred items and activities, in a free operant preference assessment (Cooper et al., 2020) in the preschool classroom. Once he or she indicated engagement with an activity by interacting with it for 10 s, a VSD was constructed by the researcher that represented the item or activity. The researcher then interrupted the activity, contriving an opportunity for the participant to mand for the item or activity. For example, if the participant was playing with crayons, the researcher created a VSD for the participant to mand for crayons and then, by restricting access to crayons, contrived an opportunity for the participant to mand for crayons. If the participant independently touched the hotspot with enough force to evoke the digitized output within three seconds, a correct mand was documented on the data sheet, and the participant was given access to the item or activity for 30 s, and the trial was considered concluded. If the participant did not mand for the item, the trial was considered over, and the next trial was presented. Baseline continued in this fashion until the introduction of intervention. Baseline lasted five sessions for Brianna and 12 sessions for Vera and Nick.

Intervention. Intervention sessions proceeded identically to baseline; however, a three-second time delay, with least-to-most prompting (i.e., model-partial physical-full-physical), was used to evoke the correct response if the participant did not independently respond within three seconds. If a prompt was needed to evoke a mand, a prompt was coded, and the participant was then given access to the item or activity for 30 s. Data were collected on whether a response was independent or prompted; however, prompt level (model, partial physical, full physical) was not recorded. At this point, the trial was considered concluded. Trials continued in this manner until the participant was given five opportunities to respond, and the session was concluded.

### 2.6. Interobserver Agreement

Interobserver agreement data were collected for 49% of all baseline (100%) and training sessions (35%), with data collected by the primary and secondary interventionists assigned to a participant during live data collection. IOA data were collected evenly across all three participants. IOA was calculated by dividing the number of agreements between two independent observers by the total number of agreements plus disagreements and multiplying by 100. IOA for baseline and training sessions was 100%.

### 2.7. Procedural Fidelity

To ensure the fidelity of the independent variable, a procedural fidelity checklist was completed following the conclusion of each baseline and intervention session. Fidelity observations occurred concurrently with sessions by trained observers. Procedural fidelity data were collected during 100% of baseline and intervention sessions. The checklist evaluated whether interventionists: (a) followed the mand training procedures as outlined, (b) implemented prompting procedures as outlined, when applicable, (c) collected data as outlined, (d) provided appropriate reinforcement following independent or prompted mands, and (e) documented whether any extraneous prompting occurred during the session. Procedural fidelity was calculated by dividing the number of correctly implemented components by the total number of applicable components and multiplying by 100. Interventionists demonstrated 100% procedural fidelity across all baseline and intervention sessions.

## 3. Results

**Figure 1:**
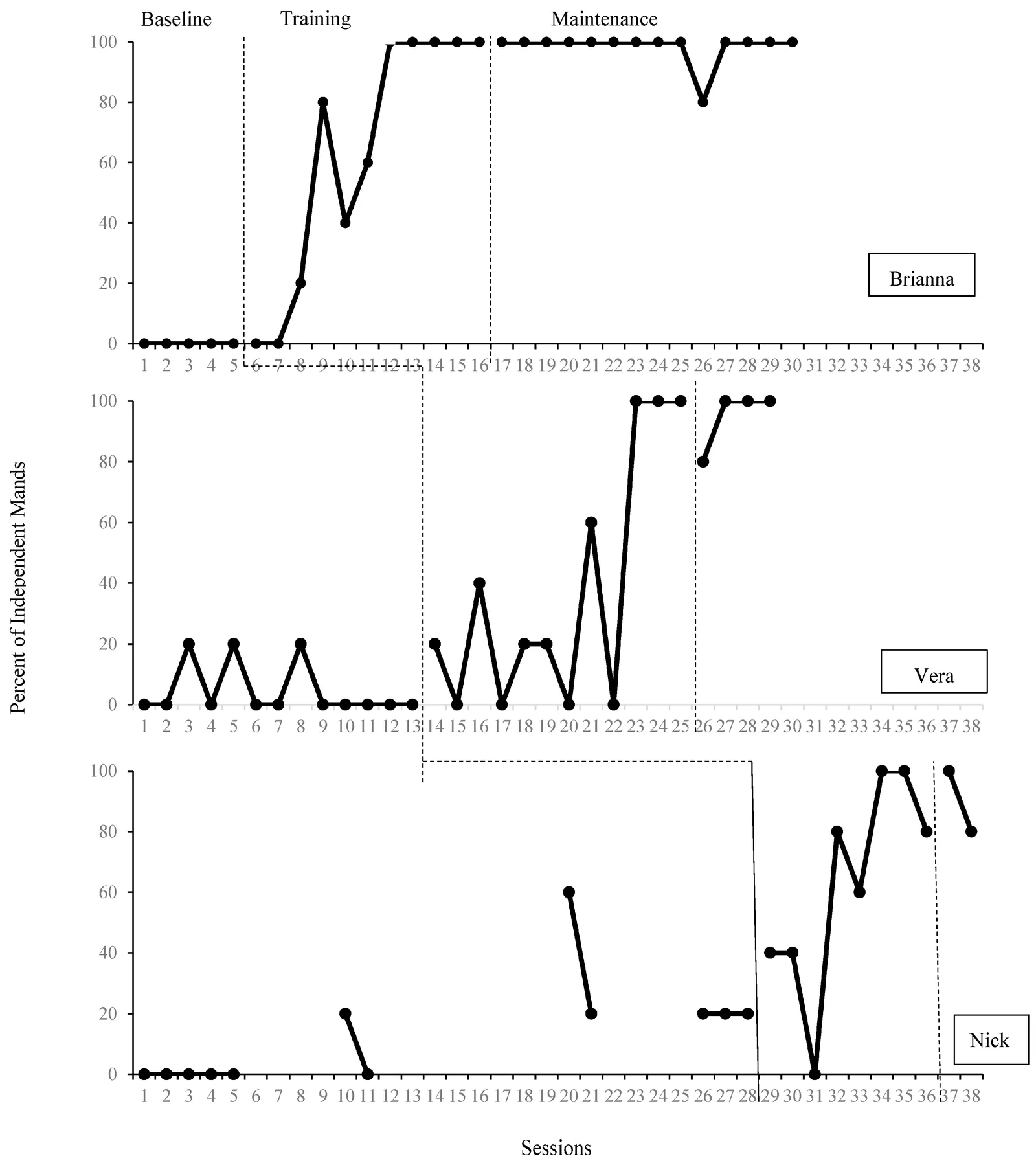
Percent of Independent Mands Across Participants. Note. This figure depicts the percent of independent mands for each participant across baseline, training, and maintenance. Within-phase breaks in data points and trendlines reflect missing sessions due to participant absences.

As shown in Figure 1, all three participants showed an increase in independent mands from baseline to intervention, with gains maintained following the achievement of mastery criteria for two of three participants. Results are presented using visual analysis, including an examination of level, trend, variability, and overlap between phases. Consistent with the logic of the non-concurrent multiple baseline design, the findings provide preliminary evidence supporting the intervention package and its association with increases in independent manding across participants. However, given Nick’s somewhat unstable baseline and overlap between phases, the results should be interpreted as proof-of-concept rather than definitive evidence of a functional relation. Each participant’s data are described individually below. For each participant, Tau-U (Parker et al., 2011) was calculated comparing baseline to intervention phases; maintenance phase data were excluded from all effect size calculations. Ninety-five percent confidence intervals were calculated for each Tau-U value using the normal approximation to the S statistic (Parker et al., 2011). Baseline data for each participant were also evaluated for trend using Kendall’s rank correlation; where a statistically significant baseline trend was present, a baseline trend-corrected Tau-U (Tarlow, 2017) was calculated in addition to the standard Tau-U.

### 3.1. Brianna

During baseline, Brianna independently manded for preferred items in 0% of opportunities across all five sessions. Brianna was the first participant introduced to the intervention following the completion of five sessions of baseline. Upon intervention implementation, Brianna’s results suggested initial skill acquisition by the third session of intervention, with independent manding increasing to mastery criteria over the course of nine sessions. Average responding during intervention was 56% with a range of 0–100% across nine sessions. Following the achievement of mastery criteria, Brianna transitioned to the maintenance phase and remained there for the duration of the study.

Visual analysis of Brianna’s data indicated a large magnitude of effect, characterized by a gradual upward shift from consistently low levels of independent manding during baseline to consistently high levels across late intervention and maintenance. Variability was low during baseline, elevated during early intervention as responding fluctuated between 0% and 80%, and subsequently decreased as performance stabilized at mastery levels. Minimal overlap was observed between baseline and intervention data points, as the first two intervention sessions remained at 0% within the baseline range; however, all subsequent intervention data points exceeded baseline levels, indicating strong differentiation between conditions. Tau-U (Parker et al., 2011) was used to determine the effect size for Brianna and was 0.88, 95% CI [0.23, 1.00], indicating a moderate to large treatment effect and supporting the visual analysis of a substantial increase in independent manding following intervention.

### 3.2. Vera

During baseline, Vera independently manded for preferred items at 5% independence with a range of 0–20% during 12 baseline sessions, with responding consistently low and steady throughout. Vera was the second participant introduced to intervention following Brianna’s achievement of mastery criteria. Upon intervention implementation, Vera’s results provide preliminary evidence of considerable variability in independent manding, with performance fluctuating between 0% and 100%, with an average of 38% across 13 intervention sessions. Following the achievement of mastery criteria, Vera transitioned to the maintenance phase, demonstrating an average of 95% independence with a range of 80–100% across four maintenance sessions.

Visual analysis of Vera’s data indicated a moderate magnitude of effect, characterized by a gradual and variable shift from low levels of independent manding during baseline to higher and more stable levels across late intervention and maintenance. Variability was moderate throughout, with fluctuating responding observed across both baseline and early intervention phases prior to stabilization at mastery levels. Overlap between baseline and intervention was moderate, as multiple early intervention data points fell within the 0% to 20% range observed during baseline. Clear differentiation between conditions emerged in final sessions of intervention and was maintained across the maintenance phase. Tau-U (Parker et al., 2011) was used to determine the effect size for Vera and was 0.61, 95% CI [0.15, 1.00], indicating a moderate to large treatment effect and supporting the visual analysis of a substantial increase in independent manding following intervention.

### 3.3. Nick

During baseline, Nick independently manded for preferred items at an average of 13% independence and a range of 0–60% independence. Baseline responding was variable, with the majority of sessions at 0%, and isolated higher responding observed at 20% and 60%. Nick was the third participant introduced to intervention following Vera’s achievement of mastery criteria. Although baseline data reflected variability with an upward trend, Nick advanced to intervention prior to achieving data stability due to time constraints, participant absence, and scheduling variables; however, the final three sessions of baseline were 20% prior to the introduction of the intervention. During the intervention phase, Nick’s results are consistent with the interpretation of variability in independent manding, with an average of 68% independence and a range of 20–100%, requiring nine training sessions to reach mastery criterion. He demonstrated initial maintenance of the acquired skill across two maintenance sessions, with scores of 100% and 80%.

Visual analysis of Nick’s data indicated a modest to moderate magnitude of effect. Increases in independent manding were observed during intervention. However, the magnitude of change was less dramatic than Brianna and Vera, given that baseline responding reached values as high as 60% across attended sessions. Variability was moderate to high across both baseline and intervention phases, with considerable fluctuations in responding observed throughout and less consistent stabilization at high-performance levels compared to other participants. Overlap between baseline and intervention phases was substantial, with several intervention data points falling within the range of baseline responding. Limited differentiation between conditions was observed until the final sessions of intervention, at which point responding reached and maintained mastery levels. Tau-U (Parker et al., 2011), comparing baseline to intervention phases only and excluding maintenance data, was 0.75, 95% CI [0.24, 1.26], which, considered alone, would suggest a large treatment effect. However, baseline data reflected a statistically significant upward trend (Kendall’s tau = 0.47), and Tau-U was therefore recalculated using a baseline trend-corrected method (Tarlow, 2017) that removes the contribution of this trend from the effect size estimate. The trend-corrected Tau-U was 0.46, 95% [−0.06, 0.99], which was not statistically significant. This corrected estimate is more consistent with the visual analysis described above, which indicated considerable overlap between baseline and intervention data and limited differentiation between conditions; Nick’s data should therefore not be interpreted as reflecting a substantial treatment effect.

## 4. Discussion

The purpose of this study was to evaluate the use of an AI-powered prototype VSD in the acquisition of a mand repertoire, when combined with a time delay, least-to-most prompting, and reinforcement for young children with CCN, either with a suspected or confirmed autism diagnosis. Overall, the findings provide preliminary evidence that the prototype may support mand acquisition. Visual analysis, supported by Tau-U effect size estimates, provides preliminary evidence of improvements in independent manding across all three participants following introduction of the intervention. While one of the participants had very rapid acquisition (i.e., Brianna), the other two (i.e., Vera and Nick) had moderately rapid acquisition requiring 9, 9, and 13 sessions (Brianna, Nick, and Vera, respectively) to reach mastery criteria. The variability observed during Nick’s baseline sessions may reflect factors unrelated to the intervention, such as practice or repeated exposure to the assessment procedures, although the specific contributors cannot be determined from the present study. This pattern underscores the need for additional replication to better understand the stability of the procedures and the extent to which the intervention contributed to observed changes. Differences in rates of acquisition across participants may also have been influenced by variability in attendance or fluctuations in motivation over the course of the study; however, these factors were not systematically measured and therefore should be interpreted with caution. That said, these results are consistent with previous research on the use of VSD with young children who have CCN, including autism.

While the results are affirmative that the prototype was effective for mand acquisition for all three participants, these results should be considered preliminary. While this prototype may address one of the calls for personalized and autonomous communication systems from adult AAC users (Lorah et al., 2022), it is hardly a complete system. Additional features must be added, and coinciding research should be conducted that includes young participants with CCN designing their own VSDs and comparisons of that process to traditional grid displays. Further considerations could also be included that involve peer perceptions and acceptance of VSDs, as peers are considered a key stakeholder group for young AAC users (Lorah et al., 2021; Light et al., 2025).

Although the findings should be interpreted cautiously, this proof-of-concept study offers several preliminary implications for clinicians working with young children with CCN. First, the findings suggest that AI-supported VSDs have the potential to reduce some of the programming demands associated with creating personalized communication displays, making contextually relevant vocabulary more readily available during naturally occurring interactions. Second, the results provide preliminary support for the continued use of highly personalized, activity-based communication opportunities to promote early mand acquisition. However, clinicians should not conclude from this study that AI-powered VSDs are superior to existing AAC systems or that AI features alone improve communication outcomes. The prototype evaluated in this study represented an early-stage technology that was implemented as part of a broader instructional package that included embedded communication opportunities, least-to-most prompting, reinforcement, and clinician support. Therefore, the observed outcomes should be attributed to the complete intervention package rather than the AI component in isolation.

From a Causal Agency Theory perspective, self-determination develops through the interaction between a person’s capacities and the opportunities, experiences, supports, and contexts available to them (Shogren & Raley, 2022). In other words, autonomy-supportive environments create opportunities for people to experience autonomy, competence, and relatedness while engaging in meaningful and self-directed action (Reeve, 2012; Ryan & Deci, 2017; Shogren & Raley, 2022). Because VSDs are grounded in personally meaningful experiences and embedded within naturally occurring contexts, they may represent one way to create autonomy-supportive communication environments for young children with CCN. By providing opportunities to communicate about activities, people, and experiences that matter to them, VSDs may support children in expressing preferences, directing interactions, making decisions, and influencing outcomes within their environments. Repeated opportunities to engage in such actions may contribute not only to communication development, but also to the development of self-determination over time (Shogren & Shaw, 2016; Shogren et al., 2019).

The current study provides a preliminary step toward this opportunity to explore how VSDs can concurrently enhance communication and self-determination development; however, it is important to note that the integration of self-determination in this research was conceptual rather than a measured outcome. As such, future research should empirically examine the potential of VSDs that are made more efficient through the integration of AI beyond mand acquisition, including their influence on engagement, participation, self-determination, and quality of life outcomes. Research should also explore opportunities for AAC users to actively participate in the creation and modification of their own communication supports, further aligning AAC technologies with principles of autonomy support, causal agency, and person-centered design. One final consideration is that the intervention package included time-delay, least-to-most prompting, and reinforcement, all of which are evidence-based practices for children with autism (Cooper et al., 2020). Therefore, it may not be surprising that the results were generally affirmative, and a comparative study is necessary to further test the utility of the prototype when compared to traditional AAC layouts.

### Limitations

Several limitations of the present study should be noted when interpreting the findings. First, participant attendance was inconsistent across all phases of the study, particularly for Nick, whose frequent absences resulted in an extended baseline phase and precluded the collection of a stable and continuous baseline prior to intervention. Inconsistent attendance also limited the number of maintenance data points collected, reducing the confidence with which maintenance of skills can be interpreted. An additional limitation was the size of the mand repertoire. Each participant only acquired mands for one to two items within the scope of the study. While this provides a basis for “proof-of-concept”, it remains unknown whether the prototype would support acquisition of larger and more diverse mand repertoires across multiple communication partners, activities, settings, and naturally occurring communication opportunities. Future research should evaluate whether AI-supported VSDs can facilitate the acquisition, maintenance, and generalization of broader communication repertoires that are meaningful for everyday participation and autonomy. Future research should also implement procedures to minimize session interruptions, such as coordinating closely with caregivers and scheduling sessions during consistent time periods. The lack of data collection on the type of prompt used to evoke the correct response also presents a limitation of the study design. An additional limitation was the absence of social validity data. Although the intervention was conceptually framed around personalized communication, autonomy, and user-centered AAC design, information regarding the acceptability, feasibility, or perceived usefulness of the prototype from participants, caregivers, clinicians, or other communication partners was not collected, as this was beyond the scope of the current study.

The VSD used in this study was a prototype and did not represent a finalized commercially available AAC application. The findings may not fully generalize to contexts in which a polished and stable version of the technology is used. Several technical limitations of the prototype were observed throughout the study and may have influenced participant performance. Specifically, the prototype suggested notable delays in hotspot creation following the capture of photographs, which interrupted the efficacy of session preparation and may have impacted motivation, as motivation may have shifted in the three seconds necessary to create the VSD. Additionally, the prototype did not include a locking mechanism to prevent participants from clearing the VSD, which introduced variability in the consistency of the visual scene display presented during trials. Further, successful hotspot activation required a degree of fine motor precision that may have posed challenges for some participants, potentially resulting in an underestimation of communicative intent. These technological limitations highlight the preliminary nature of the current investigation and underscore the need for further development and refinement of the AI-powered VSD generator prior to broader clinical implementation. Finally, comparisons between this prototype and traditional grid displays are necessary to further the benefits of the VSD.

## 5. Conclusions

Despite these limitations, this proof of concept provides positive results that a personalized VSD, combined with model prompting, can promote manding in young autistic children with CCN. This study represents an early step towards AI-supported AAC features that may enable greater customization for user-selected vocabulary. However, additional work in this area is needed, and these results should be interpreted with caution. Future research should extend these findings by offering additional opportunities for personalized and context-aware communication systems, as is requested by adult AAC users (Lorah et al., 2022). In sum, personalized communication supports that are responsive to a person’s interests, experiences, strengths, and current environments may not only increase communication opportunities, but can also create more autonomy-supportive contexts in which children can express preferences, direct interactions, and influence outcomes that matter to them. As the intersection of AAC and AI continues to evolve, there is potential to design systems that support both communication development and the broader development of agency, participation, and self-determination across the life course (Holyfield et al., 2025; Light et al., 2025; McNaughton et al., 2025).

## Figures and Tables

**Table 1:** Participant Information.

Name	Sex	Age	Race/Ethnicity	VB-MAPP Score
Nick	M	3.0	White/Caucasian	20.5
Brianna	F	4.7	Hispanic	26.5
Vera	F	2.8	White/Caucasian	29.0

Note. Age is reported in years. VB-MAPP = Verbal Behavior Milestones Assessment and Placement Program (Sundberg, 2008).

## Data Availability

The authors affirm that participants and/or their legal guardians gave written informed consent for the inclusion of the data within this article. All data generated from this study were included in the current manuscript.
